# Integrity Verification of Distributed Nodes in Critical Infrastructures

**DOI:** 10.3390/s22186950

**Published:** 2022-09-14

**Authors:** Silvia Sisinni, Davide Margaria, Ignazio Pedone, Antonio Lioy, Andrea Vesco

**Affiliations:** 1 Department of Control and Computer Engineering, Politecnico di Torino, 10129 Torino, Italy; 2 LINKS Foundation, 10138 Torino, Italy

**Keywords:** trusted computing, remote attestation, global navigation satellite system, 5G networks, precision time protocol

## Abstract

The accuracy and reliability of time synchronization and distribution are essential requirements for many critical infrastructures, including telecommunication networks, where 5G technologies place increasingly stringent conditions in terms of maintaining highly accurate time. A lack of synchronization between the clocks causes a malfunction of the 5G network, preventing it from providing a high quality of service; this makes the time distribution network a very viable target for attacks. Various solutions have been analyzed to mitigate attacks on the Global Navigation Satellite System (GNSS) radio-frequency spectrum and the Precision Time Protocol (PTP) used for time distribution over the network. This paper highlights the significance of monitoring the integrity of the software and configurations of the infrastructural nodes of a time distribution network. Moreover, this work proposes an attestation scheme, based on the Trusted Computing principles, capable of detecting both software violations on the nodes and hardware attacks aimed at tampering with the configuration of the GNSS receivers. The proposed solution has been implemented and validated on a testbed representing a typical synchronization distribution network. The results, simulating various types of adversaries, emphasize the effectiveness of the proposed approach in a wide range of typical attacks and the certain limitations that need to be addressed to enhance the security of the current GNSS receivers.

## 1. Introduction

Several infrastructures require accurate time distribution and synchronization, e.g., energy grids, transport and telecommunication networks, all with different requirements in terms of synchronization accuracy and precision [1,2,3]. Some of these infrastructures are classified as critical infrastructures, so they must be properly protected against cyberattacks.

According to the Council of the European Union, a critical infrastructure is an asset, system or a part with functions that are essential to society, the economy, safety, security and well-being of people, whose failure or disruption would have a significant impact on those functions [4]. The 5G network has the potential to quickly become the infrastructure on which the operation of national and international critical infrastructures will be based, redefining the way in which transportation, medical and emergency services, agriculture, water distribution, energy grids and many other vital sectors will be managed; in such a context, it can be considered the most critical of the critical infrastructures. 5G network can connect a huge number of devices, up to 1 million per square kilometer, which, on the one hand, brings enormous computational and storage capacity to the edge of the network, enabling the creation of bandwidth- and latency-sensitive applications that were previously impossible to realize; on the other hand, it leads to an increase in attack surface available to cybercriminals, who can exploit many more entry-points from which they can penetrate the network. For this reason, it requires a detailed analysis of the risks to which it is exposed and specific security architectures.

5G technology poses unprecedented challenges, requiring the management of an accurate and reliable synchronization among multiple timing sources across the overall network. In addition, highly accurate time, frequency and phase synchronization are needed at different hierarchical levels of the network architecture to realize the full potential of 5G, including the benefits of new Time Division Duplex (TDD) spectral efficiency. There is also a need for increased reliability of the timing sources; while nowadays, Long-Term Evolution-Frequency Division Duplex (LTE-FDD) networks can continue operating for hours after losing the synchronization without significant degradation, in the future 5G networks, a loss of timing will have an immediate impact on Radio Access Network (RAN) performance [5].

In this context, satellite-derived timing information plays a key role in the provisioning of an absolute time reference to current and future telecommunications networks. *Global Navigation Satellite System* (GNSS) receivers combined with terrestrial caesium clocks and specific transport protocols can indeed satisfy the stringent requirements foreseen in 5G networks by granting sub-nanosecond synchronization accuracy [3]. The importance of time synchronization in 5G networks makes the time distribution network a likely target for potential attacks, so it is essential to adopt appropriate countermeasures. In fact, possible synchronization inaccuracies between clocks can directly result in a degradation of the quality of service provided by the 5G data network (e.g., reduced throughput, increased latency and jitter) and, in extreme cases, a complete disruption of the 5G service.

In the recent scientific literature, several categories of attacks have been analyzed, with particular regard to those against the GNSS Radio-Frequency spectrum and the timing protocols over the network, and appropriate countermeasures have been proposed (e.g., refer to [6,7,8] and references therein). In *Trusted GNSS-Based Time Synchronization for Industry 4.0 Applications* [9], the authors analyzed the attacks that compromise the integrity of software and configurations in the nodes involved in the synchronization distribution network and proposed a framework to monitor the integrity of nodes and detect tampering with them.

This work addresses, in addition to the attacks considered in [9], “openbox” hardware attacks aimed at modifying the configuration set on the GNSS receiver, not yet investigated to the best of the authors’ knowledge. In order to also detect this kind of violation, we propose a solution based on Trusted Computing technologies and present a possible implementation. The main contributions of this paper are:

- a remote attestation architecture with a modified workflow to monitor the current configuration of the GNSS receiver;
- the integration of a new attestation workflow in Keylime, a Cloud Native Computing Foundation-backed remote attestation framework supporting both TPM 2.0 specifications and the Integrity Measurement Architecture (IMA) Linux security module;
- the analysis and evaluation of the proposed approach by leveraging an effective testbed.

The rest of the paper is organized as follows: Section 2 summarizes the various categories of attacks against time distribution networks and describes the threat model considered. Section 4 reviews the Trusted Computing technologies adopted in this work. Section 4.4 presents the architecture of the solution we propose and Section 6 describes, at a high level, its implementation. Section 7 presents the testbed used for the experimental tests and describes the types of simulated attacks, while Section 8 analyzes the results obtained from them. Finally, Section 9 concludes the paper with the key aspects that emerged from this work, and it highlights the challenges to be faced in future studies.

## 2. Related Works

In recent years, researchers have proposed various schemes that aim to evaluate the trustworthiness of sensing data in order to increase the security and reliability of systems by relying on diversified techniques.

Jiang et al., [10] studied the threats related to cyber-physical attacks in critical infrastructures and proposed an integrated data-driven framework to protect the industrial data from eavesdropping and integrity attacks. The protection from eavesdropping attacks is achieved through the offline design of encryptor and decryptor modules; the encryptor module superposes data measurements and check codes to auxiliary signals to encrypt data before transmission over the network, while the decryptor module recovers the sensor measurements at the receiver end. The solution comprises also a “trustworthiness judgement” module, designed to detect the manipulation of data with a high degree of sensitivity and to decide if the received data have a sufficient level of trustworthiness to be used for monitoring and control purposes. The proposal is an interesting solution for the protection of data in transit on the network; however, it does not cover attacks aimed at compromising the nodes that generate data.

Ren et al., [11] define a privacy-protected intelligent crowdsourcing scheme based on reinforcement learning (PICRL), an innovative solution aiming at optimizing the utility of crowdsourcing applications. The maximization of the utility of the system is achieved through the evaluation of the data quality, which is obtained by rating the trustworthiness of each participant to the system. The proposed trust evaluation mechanism consists of three phases which complement each other: privacy trust, crowd trust and hybrid active trust. This is an interesting solution as it aims to assess the trustworthiness of unknown data sources; however, due to the characteristics inherent in the system, the trustworthiness evaluation of nodes is probabilistic. Our work, instead, through Trusted Computing techniques, aims at a deterministic evaluation of the trustworthiness level of computational nodes that are part of a 5G network by evaluating the integrity status of their entire software stack and the configuration set on them.

Guo et al., [12] focus their attention on sensor-cloud systems (SCSs) and propose a solution to evaluate the credibility of Mobile Vehicles (MVs), which act as data collectors and transfer data from local IoT sensing devices to the cloud for further processing. This solution estimates the trustworthiness of the MVs through two types of calculations: (a) Direct trust calculation, in which the evaluation of a MV is carried out by comparing the data sent by it to the cloud with reference data acquired through Unmanned Aerial Vehicles (UAVs), or by verifying whether the sent data contains a code that guarantees its authenticity; (b) Trust inference calculation, in which the credibility of a MV is estimated by comparing its data with those sent by MVs deemed trusted by the system. In this scheme, the IoT devices installed in the smart city are considered trusted by design, and the work focuses on a probabilistic estimate of the reliability of unknown data collectors; in our work, instead, no node within the infrastructure is considered trusted a priori.

Mo et al., [13] take into consideration the reliability of Edge Computing applications, whose decisions are based on data forwarded by sensing IoT devices. In this kind of architecture, typically many devices are self-organized in IoT networks to send the sensed information to data collection nodes at the center of the network—the so-called sink nodes; these, in turn, forward collected data to mobile vehicles in order to exploit their capability to connect directly with the Internet to send data to the cloud. A feature of these networks is that IoT devices transmit their data to sinks through multi-hop routing and, since IoT networks are typically open networks, malicious IoT devices can be added in order to alter the correct sending of data, causing the application to take wrong decisions. Typical attacks are the “black hole”, where malicious nodes drop all packets in order to stop the data collection, and the “Selective Forwarding Attack” (SFA), which is more insidious than the first one because it is more difficult to detect, in which malicious nodes selectively drop some packets to protect themselves from being discovered. The work of Mo et al., proposes a solution to assess the credibility of IoT nodes as trustworthy forwarding nodes, in order to ensure a reliable and low-cost data collection. This work, similar to the previous ones, does not take into consideration Trusted Computing techniques for evaluating the reliability of nodes; furthermore, the solution does not cover attacks aimed at compromising the sources of information, which is the focus of our work.

With this work, we began to explore the feasibility of applying Trusted Computing techniques to monitor the integrity of nodes that are part of a network, thus, enhancing the security and reliability of critical infrastructures that are similar to today’s 5G networks.

## 3. GNSS-Based Time Distribution Networks

In recent years, GNSS technologies [14,15] have been experiencing a remarkable development, both concerning global constellations and users’ receivers. Nowadays, they are leading positioning and synchronizationtechnologies and are adopted in several fields, such as 5G networks, thus, representing the use case discussed in this paper.

**Figure 1:**
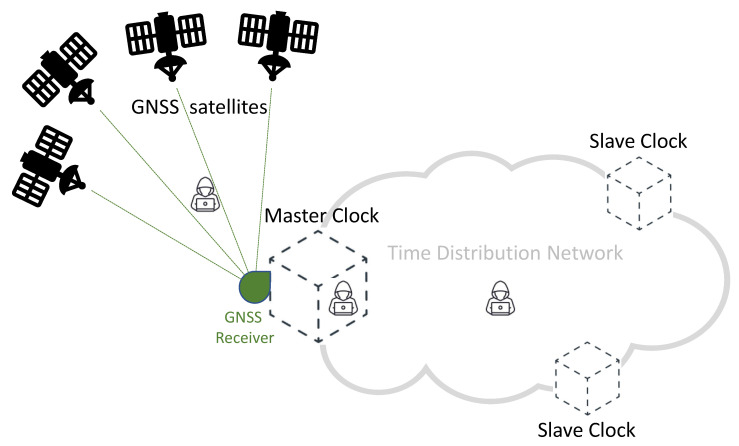
Simplified scheme of a GNSS-based time distribution network.

Figure 1 provides a simplified diagram of a synchronization network, representing a typical telecommunication network and highlighting the role of GNSS technologies. In this diagram, accurate time information is generated by each GNSS satellite, and then transmitted by the means of adequately formatted Radio-Frequency (RF) signals. A Master Clock node of the network accurately synchronizes its local clock using a GNSS receiver. As illustrated in Figure 1, a distribution network allows distributing timing information to all the nodes of the network, achieving an accurate synchronization between the Master Clock and multiple Slave Clocks. Different protocols and technologies can be adopted on the distribution network, depending on the application requirements and constraints. Among others, the *Precision Time Protocol* (PTP) represents a well-known and widely adopted solution for accurate time distribution in a network of clocks organized in a master–slave hierarchy [3]. Such synchronization protocol is also known as IEEE-1588 standard [16,17]: it allows for absolute time synchronization in the range of hundreds of nanoseconds through hardware assistance (SyncE), and potentially, with sub-nanosecond accuracy with the White Rabbit extension of PTP (WR-PTP) [18,19].

The *GNSS RF spectrum* and the *Time Distribution Network* are two areas identified as critical in Figure 1: they both represent viable attack vectors, thus, due to the importance of synchronization in 5G networks, they need appropriate protection against potential attacks.

GNSS RF spectrum attacks can be classified in three main categories [1,7,8,9]:

- *Jamming*, the blocking of the reception of GNSS signals by intentionally introducing a powerful RF signal to overwhelm the signal used by the receiver; this threat is classified as a “Denial of Service” (DoS) attack, since it denies the service to all nodes within the interference range;
- *Meaconing*, this corresponds to the interception and rebroadcasting of GNSS signals on the same transmission frequency, typically with a higher power than the original signal, in order to confuse the data acquired by the victim receiver;
- *Spoofing*, this refers to the transmission of counterfeit GNSS-like signals with the intent to fool the victim receiver with a false position and/or time data.

The GNSS research community has extensively discussed and made available possible countermeasures against these attacks; interested readers can learn more about these topics by reading [7,8,20] and the references therein.

Time Distribution Network attacks can, in turn, be grouped into two categories:

- attacks targeting *PTP over the network*, such as packet content manipulation, packet removal, packet delay manipulation and packet replay; these attacks are categorized as DoS and “Man-In-The-Middle” (MITM) attacks;
- attacks against the *integrity of PTP* (both software and configuration) running on each node.

In the scientific literature, we can find many examples of solutions and mitigation techniques against attacks on PTP over the network: a new version of the IEEE-1588 standard, containing a security extension based on a multi-pronged approach [17]; a new key management mechanism for PTP, used in Network Time Security (NTS) [21]; the “First Packet Authentication with Transport Access Control” [6], an identity-based authentication system. Regarding the attacks against PTP integrity, they were analyzed in [9], where the authors proposed a solution based on Trusted Computing technologies in order to detect tampering with PTP software and configurations set on the nodes.

### Threat Model

Our work aims to propose a solution to detect and mitigate attacks against PTP integrity. The nodes involved in the time distribution network can be exposed to hardware and software attacks by malicious adversaries:

- a *hardware adversary* can physically tamper with the host’s System on Chip (SoC) or other hardware devices present on the board, such as the *Trusted Platform Module* (TPM) or the GNSS receiver, and can interfere with the communication among them by injecting unauthorized signals on platform buses;
- a *software adversary* can remotely take control of the nodes in order to infect them with malware, modify their executable and configuration files, and corrupt portions of the host’s RAM.

For instance, an adversary may locally perform a hardware bus hijack attack to compromise data integrity and confidentiality between two endpoints that communicate on the board, such as the GNSS receiver and the host CPU. Moreover, an adversary may remotely tamper with the software and the configurations on the host system. In this work, we assume that all nodes are equipped with trusted hardware (i.e., TPM chip) and that load-time integrity measurements can detect unauthorized changes to the software and configurations on the host system. We do not consider runtime memory attacks, which can be mitigated through protection mechanisms offered by modern operating systems, such as address space layout randomization [22].

## 4. Trusted Computing: Motivation and Technologies

This section gives a brief overview of some concepts related to Trusted Computing, Trusted Platform Module, and Remote Attestation. Moreover, we introduce some well-known techniques to perform periodic remote attestation of distributed nodes that leverage the Integrity Measurement Architecture module of the Linux kernel.

### 4.1. Trusted Platforms

The concept of *trust* in cybersecurity concerns the determination of the behavior of an entity: it is trusted if it behaves as expected for the intended purpose; consequently, it is trustworthy if its behavior is predictable [23]. The problem of establishing the trustworthiness of a platform is difficult to solve purely through software solutions. This is because, if a platform has been compromised, the software will lie about its actual integrity state, generating a sort of “circular” problem, as we can trust the software’s response about its integrity state only if the software is trusted [24]. In the early 2000s, the *Trusted Computing Group* (TCG) addressed this problem by proposing a hardware anchor on which to build trust in a platform, the *Trusted Platform Module* (TPM). This anchor was a secure cryptoprocessor that was physically attached to the motherboard of the device and defined the guidelines for creating *Trusted Platforms* (TPs).

A TP is a platform which employs sufficient hardware and software integrity measurements and is able to collect and report them to third parties in a way that permits to determine whether their behavior is the expected one. Trust in a platform is founded on the so-called *Roots of Trust* (RoTs), defined by the TCG as the set of system elements inherently trusted, secure by design, and whose misbehavior is not detectable at runtime [23]. In order to establish the trustworthiness of a platform, the TCG requires that it provides, at a minimum, three RoTs: a *Root of Trust for Storage* (RTS), a *Root of Trust for Reporting* (RTR) and a *Root of Trust for Measurement* (RTM).

The RTS is represented by a set of memory locations whose access and modification can only take place in a protected and secured manner. The TPM implements the RTS through its memory locations, defined as *Shielded Locations*, since they can be accessed by external entities only through *Protected Capabilities*, enforced by the TPM to protect the objects from disclosure, tampering and deletion without authorization. Several shielded locations are used to store sensitive information, such as the private part of asymmetric keys. Other locations, such as the so-called *Platform Configuration Registers* (PCRs), are used for storing integrity measurements of platform components—integrity measurement being the digest computed through a cryptographic hash function on the software and the configurations of the platform—and their values can be modified only through *reset* or *extend* operations. The *reset* operation sets the PCR value to its default initial condition, which is either all bit zero or all bit one; it occurs at power-on of the platform and can be performed afterwards only if allowed by an attribute of the PCR. The *extend* operation is used to cumulatively aggregate an indefinite number of integrity measurements into a single PCR, overcoming the problem of the limited number (typically 24) of PCRs available in the TPM, according to the following equation:

(1) $$PCR_{new}=H_{hashAlg}(PCR_{old}\;||\;measure_{new})$$

where each term of the equation means:

$PCR_{new}$: the new value of the PCR after the *extend* operation;
$H_{hashAlg}$: cryptographic hash algorithm associated with a specific PCR bank;
$PCR_{old}$: the value of the PCR before the *extend* operation;
$measure_{new}$: a new integrity measurement that is concatenated to $PCR_{old}$ value and the resulting concatenation is hashed to produce the result of the *extend* operation.

The order of the measurements extended in a PCR is also preserved due to the irreversibility property of cryptographic hash algorithms.

The RTR provides the capability to report on the contents of the RTS, with the exception of locations containing sensitive information. The TPM implements the RTR because it has the cryptographic capabilities to create a report, which is a digitally signed digest computed on the values of some shielded locations (such as PCRs, audit logs, properties of keys), and is capable of giving the report a hardware-rooted authenticity based on the *Endorsement Key* (EK). The EK is a non-migratable asymmetric key, derived from a seed statistically unique for the TPM and certified by the TPM manufacturer. In order to not expose the private part of this key, the EK acts as a *Storage Key*, which is a key that protects other keys. EK is used only to decrypt certificates of other non-migratable keys, such as the Attestation Keys (AKs). AKs are TPM-generated keys used to sign the reports. Through the *quote* operation, the TPM creates an unforgeable report on a set of PCRs, to which an external entity obtains evidence of the platform configuration and can evaluate its trustworthiness.

The RTM represents the starting point of a chain of trust on which the integrity measurement process of the entire platform is founded; it is responsible for taking integrity measurements on the platform components and sending them to the RTS. The RTM is not implemented by the TPM, since this is conceived as a slave device receiving commands; it relies on an immutable piece of code, the *Core Root of Trust for Measurement* (CRTM), which represents the first set of instructions that receive control of the system at startup. The CRTM can be implemented either through CPU instructions embedded in the specific generation processors or as a small portion of code within the UEFI/BIOS. The CRTM purpose is to record, in PCR0, the BIOS version that is being used to boot the system before passing control to the full BIOS. Thus, the RTM resides in the CPU when this is controlled by the CRTM [23]. The integrity status of the platform is obtained through measurements performed by a software component on the next one that will take control of the system according to the concept of *transitive trust*: the trust in a component is the foundation upon which to build trust in the next one. The acquired measurement is extended in a PCR before the control is transferred to the measured component. This process guarantees that, even if a component is compromised, the latter cannot avoid being measured before its execution. Moreover, the irreversibility property of cryptographic hash functions ensures that the tampered component will not be able to take the extended measure out of the PCR or find a combination of extensions to forge an ad hoc value for the PCR in a computationally feasible time. This measurement procedure, repeated in a chain by all components involved in the booting sequence, realizes the so-called *Measured Boot* or *Trusted Boot*. Dissimilar to *Secure Boot*, which stops the booting process if the signature of a component is invalid or its hash value has a mismatch with a reference value, Trusted Boot does not prohibit the system from booting in an insecure state, since no verification is performed during the boot. In order to extend the chain of trust from the BIOS up to the application layer, the Linux kernel provides the *Integrity Measurement Architecture* (IMA) module, which measures all the executables, configuration files and kernel modules loaded at runtime, and extends the measurements in a specific PCR, typically in PCR10. IMA is part of the Linux Integrity Subsystem starting from kernel version 2.6.30, and is currently one of the most accepted TCG-compliant solutions for measuring dynamic executable contents [25].

### 4.2. TPM 2.0

TPM 2.0 is the latest version of the TPM specification, released by TCG in 2015 [26]; it adds numerous design goals to those inherited from the previous TPM 1.2 specification. TCG designers began to work on the new specification when the first significant attack on the hash algorithm SHA-1, heavily used in TPM 1.2, was published, so they decided to create a specification that was *agile* with respect to cryptographic algorithms, considering the common axiom in cryptography, according to which algorithms become weaker over time, never stronger [27]. Whereas TPM 1.2 allowed only specific algorithms to be implemented, with SHA-1 as the hash algorithm and RSA as the asymmetric algorithm, TPM 2.0 does not enforce the use of specific cryptographic algorithms, but it refers to another document, the *TCG Algorithm Registry* [28], containing the list of algorithms allowed by TCG, so that they can be easily upgraded to stronger ones without the need to develop a new specification. This feature is known as *Algorithm Agility* and allows the presence of multiple PCR banks—the set of the PCRs extended with the same hash algorithm—in the TPM. Another significant improvement in TPM 2.0 was the introduction of symmetric encryption for protecting user data, thus, making the process of loading private keys into TPM 2.0 much quicker than in TPM 1.2. Other improvements compared to the previous 1.2 specification concern a new TPM object authorization scheme: the *Enhanced Authorization*. This provides a more flexible management of the TPM due to the creation of distinct object hierarchies and additional capabilities to enhance the security of platform services [26].

### 4.3. Integrity Measurement Architecture (IMA)

IMA [29] is the Linux kernel’s implementation of the integrity measurement system conceived by the TCG and it extends the principles of Trusted Boot and Secure Boot to the application layer, allowing an external entity to verify not only that the platform booted in a trusted way, but also that the applications and the kernel modules loaded at runtime are trusted. The design of the IMA module includes the following major components:

- *IMA Measurement* extends the Trusted Boot principles into the Linux kernel; it is responsible for determining the files to be measured, performing measurements on them and maintaining those measurements in a secure way;
- *IMA Appraisal* extends the Secure Boot principles into the Linux kernel; it is responsible for locally comparing file measurements against trusted values stored in the file’s security extended attributes, denying access to files in case of measurement mismatch;
- *IMA Audit* is responsible for including IMA-specific records in the system audit logs, used to enhance system security analytics/forensics [29].

In this work, we used the services of IMA Measurement, which processes all files accessed at runtime according to the rules specified in the IMA policy configured on the system. The policy can be set to one of the IMA built-in policies that allow measuring the *Trusted Computing Base* (TCB) of the system or to a custom policy created according to the needs of a specific context. The files are measured by computing a digest over their complete contents, using the SHA-1 algorithm by default or the one specified by the means of a kernel command line parameter. All the measurements are kept in a *measurement list* in the kernel memory and are subsequently recorded in two log files located in the security file system, ascii_runtime_measurements and binary_runtime_measurements, so that the measurements are available in user space.

**Figure 2:**
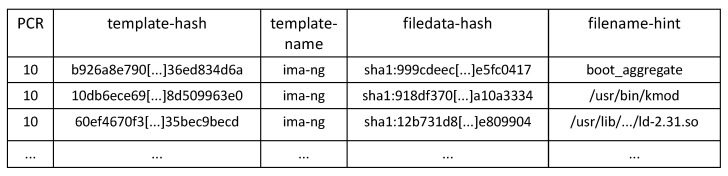
Excerpt of IMA Measurement Log according to ima-ng template.

Each node of the list can contain, in addition to the measurement of the file content and the file path, other metadata related to the measurement event, according to the configured IMA template. The IMA module provides a set of built-in templates, such as ima, ima-ng (default), ima-sig. When a new node is added to the measurement list, IMA computes a digest on all fields specified by the template, and if the platform is equipped with the TPM chip, it extends the digest in a PCR, which, by default, is PCR 10. This process makes all the following modifications to the measurement list visible to an external entity that has to verify the integrity status of the platform. Moreover, the *extend* operation is performed before the measured component takes control of the platform, either directly as executable or indirectly as a data file, so that a potentially corrupted component cannot hide its presence on the system. Figure 2 shows some entries of the IMA Measurement Log (ML) file created with the ima-ng template: from left to right, in each entry, we find the PCR used for the extend operation, the digest (computed with SHA-1 algorithm) extended in the PCR, the name of the template used, the digest computed on the file contents and the path of the measured file.

### 4.4. Remote Attestation

The *Remote Attestation* (RA) is a challenge/response protocol in which a remote entity, the Verifier or Challenger, is able to determine the trustworthiness level of a computational node, the Attester or Prover. During this protocol, the attesting system creates an authenticated and unforgeable proof of its integrity state, reliable even when the platform has been compromised, and provides it to a remote party which is able to make decisions based on that evidence and to establish whether the attesting platform has a sufficient level of trustworthiness to be used in a given environment. A generic attestation schema involves three main phases [30]: (1) hltextitChallenge, in which the Verifier creates an unpredictable nonce, used to check the freshness of the integrity evidence and avoid replay attacks, and sends it to the Attester; (2) *Attest*, in which the Attester collects the attestation data, signs it with a key identifying the platform and sends the signature to the Verifier as proof of the platform state; (3) *Verify*, in which the Verifier checks the signature received from the Attester and takes a decision about its current integrity status.

Among the various types of remote attestation that can be implemented, in this paper, we will consider an explicit attestation based on TPM 2.0 [31]. The Attester platform must be equipped with a TPM 2.0 chip, which provides an EK key with the corresponding certificate issued by the TPM manufacturer, representing the root of the chain of trust for the attestation keys (AKs). To enable RA, the Attestation Agent running on the remote platform must have the right permissions to use an AK, which can be either the Initial Attestation Key (IAK) certified by the platform manufacturer to create a binding with the identity of the device, or a Local Attestation Key (LAK), generated by the owner or administrator of the device. The Verifier has to acquire the AK certificate either through a Privacy Certificate Authority (CA) or the platform itself; moreover, it has to be able to access the reference whitelist of the attesting system. The whitelist is the set of known-good values, which are compared with the platform’s integrity measurements during the attestation process; it is provided and validated through certificates by the platform manufacturer and the software vendor, or it is generated by the system administrator, in an isolated environment the first time the platform is booted.

**Figure 3:**
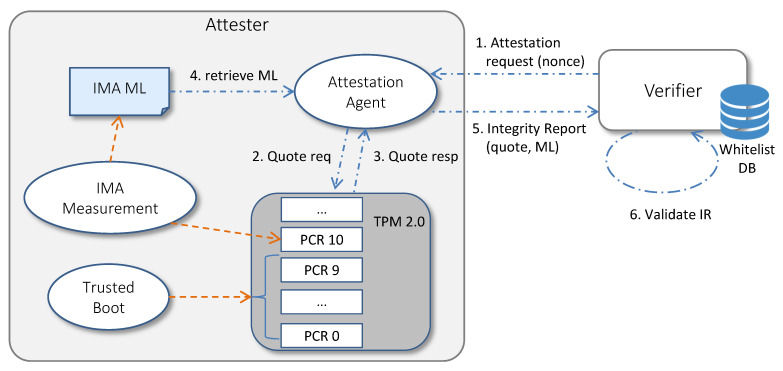
IMA-based Remote Attestation scheme.

Figure 3 shows how the TPM chip, the IMA measurement mechanism and the Trusted Boot sequence enable the RA process. (1) The RA cycle starts when the Verifier sends an attestation request to the Attestation Agent, specifying a non-predictable *nonce* and the list of PCRs to be included in the quote (typically, PCRs related to Trusted Boot and IMA). (2) The Attestation Agent sends a quote request to the TPM, indicating the received nonce and the list of PCRs. (3) The TPM responds to the quote request by loading the AK key and using its private part AK_priv_ to sign the hash of the selected PCRs, the nonce and other TPM metadata. (4) The Attestation Agent retrieves the IMA ML. (5) Then, it packages the quote, the PCR values, the IMA ML and the AK credential (that is the AK_pub_ and its certificate signed by the Privacy CA) as an Integrity Report (IR), following the TCG Integrity Reporting Schema [32], and sends the IR to the Verifier. (6) Upon receipt of the IR, the Verifier checks that the quote is fresh and authentic, the platform is booted in a trusted way, the IMA ML is non-tampered and the runtime measurements contained in it are representative of a trusted system.

The steps performed by the Verifier for validating the IR are typically the following: First, the Verifier checks the authenticity and the freshness of the quote by verifying that: the signature is valid, that is, it has been performed on the received data with the private part of the certified AK, and the AK_pub_ has not been revoked by the Privacy CA; the signed data contains the nonce sent by the Verifier in the request; the hash of the PCRs contained in the quote matches with the list of received PCRs. Then, the Verifier checks the boot of the platform by verifying that the aggregate values contained in the PCRs related to Trusted Boot match the reference golden values. Moreover, the Verifier checks the integrity of the IMA ML by walking through its entries and recomputing the PCR aggregate, as performed by the TPM—that is, by performing the extend operation with the template-hash field of each entry: if the recomputed aggregate matches the IMA PCR signed in the quote, the received ML is authentic. Finally, the integrity state of the platform at runtime is evaluated by comparing each measurement contained in the IMA ML with the known-good values contained in the whitelist.

## 5. Design of the Solution

The degradation of synchronization between nodes of 5G networks has a nefarious impact on the functioning of the network. The deployment of a state-of-the-art network, which applies all the attack protection techniques analyzed in the literature [3,6,7,8,17,21], does not guarantee the reliability of the synchronization in the event that a malicious insider manages to compromise the integrity of the software stack or the configuration of the network nodes. As a result, early detection of attacks on node integrity at any level of the network hierarchy is of paramount importance in this scenario. Remote Attestation based on the Trusted Computing principles is a security technique which allows this verification process to take place.

To address the problem of the reliability of the PTP protocol, we propose a new design of the RA protocol to monitor the integrity of all components of the network, including the configuration of the GNSS receiver, in order to establish trust in the correct synchronization of nodes.

### 5.1. Configuration of Nodes in a PTP Network

**Figure 4:**
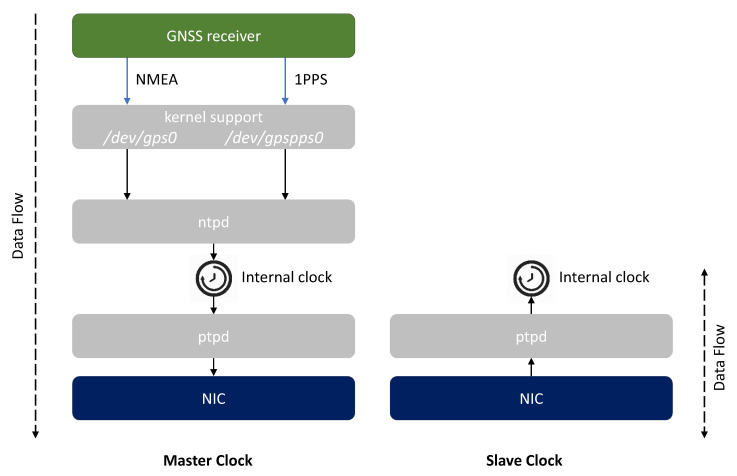
Software stack and configurations for Master Clock and Slave Clock nodes.

The integrity of the PTP protocol depends on the integrity of all software components, present on the nodes of the network, which affect its correct operation. Figure 4 represents a possible software stack and configuration applied to Master Clock and Slave Clock nodes.

A Master Clock always requires the presence of a GNSS module in order to receive the RF signals from one or multiple satellite constellations (e.g., GPS, Galileo, GLONASS, BeiDou) and derives Position, Velocity, and Time (PVT) information from them.

The GNSS receiver provides the PVT data to the host system through two interfaces:

- a textual interface over a serial communication protocol, which provides PVT data coded according to the *National Marine Electronics Association* (NMEA) 0183 standard [33];
- a 1 *Pulse-Per-Second* (1PPS) interface, which provides a high precision analog signal, with a width of less than one second and a rising or falling edge that is accurately synchronous with the beginning of each second of the time scale.

The Linux kernel offers drivers the opportunity to make these interfaces accessible as devices for the application layer.

The ntpd [34] daemon accesses the data provided by these interfaces by the means of two symbolic links to the actual devices (i.e., /dev/gps0 and /dev/gpspps0, respectively) and uses them to accurately synchronize the internal clock with respect to the GNSS time scale.

Then, the ptpd [35] daemon is in charge of distributing the time of the internal synchronized clock to the Slave nodes over the network, achieving microsecond-level time synchronization, even on platforms with limited resources.

A Slave Clock has a more straightforward software configuration: the ptpd daemon running on it, set with PTP_SLAVE status, receives the messages sent by the Master Clock and starts to correct its internal clock according to the information contained in them. Typical time distribution networks can require a remarkable amount of time (e.g., ranging from a few minutes up to several days) to achieve an initial synchronization between multiple nodes; in fact, minor frequency and phase corrections allow to gradually and continuously discipline the clocks.

From the above, it emerges that the software integrity of the PTP protocol is strictly dependent on the integrity of the kernel, which can be measured through the Trusted Boot process, and of the ntpd and ptpd daemons, whose executables and configurations can be measured by the means of the IMA module, configured with a suitable policy.

Moreover, the proper functioning of the PTP protocol also depends on the configuration set in the GNSS receiver, which is the source of information for the daemons running on the Master Clock, so it is essential to ensure that it has not been compromised at a given point in time. A physical adversary may, in fact, carry out a hardware bus hijack attack in order to inject messages that alter the correct configuration, causing synchronization imprecision between clocks. The IMA module cannot intercept such tampering since it is performed on a hardware device external to the host system, so a verifier would not be able to detect this kind of violation during the attestation process. In this paper, we propose an extension to the RA workflow in order to address this problem.

### 5.2. Solution Architecture

**Figure 5:**
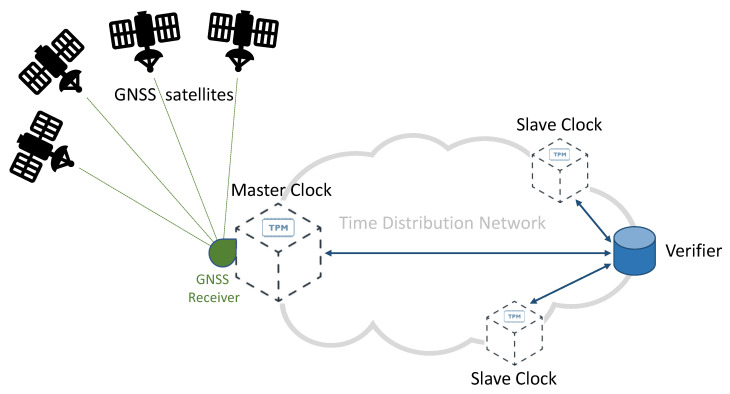
Architecture for Remote Attestation on the time distribution network.

The RA schema presented in this paper periodically certifies the integrity of all nodes within the time distribution network, as shown in Figure 5. Our goal is to monitor the firmware, the operating system and the software components running on Master and Slave nodes. We also want to check the current configuration of the GNSS receiver on the Master Clock. The attestation workflow presented in Section 4.4 can be easily used to verify the integrity of the software running on the Slave nodes. Nevertheless, in order to allow the monitoring of the GNSS receiver configuration within a RA cycle, it is necessary to make some changes to the operations performed by the Attestation Agent running on the Master Clock.

**Figure 6:**
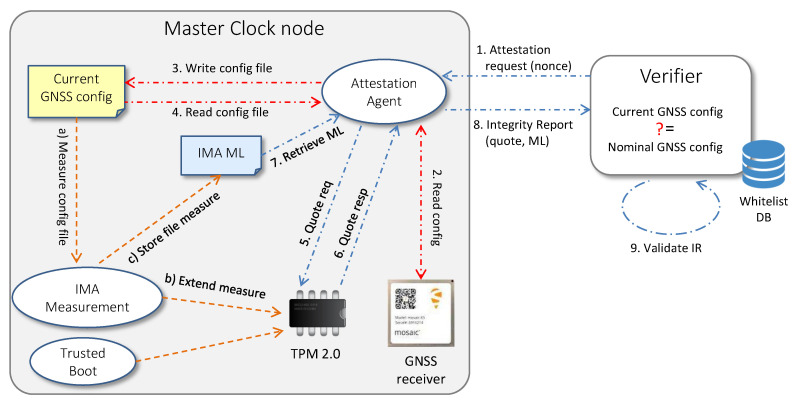
Workflow for remote attestation of a Master Clock node.

**Figure 7:**
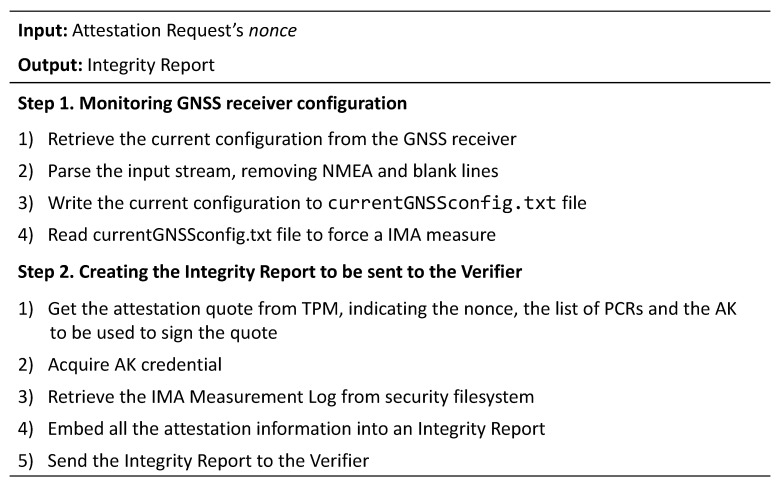
Algorithm steps of the Master Clock node.

Figure 6 depicts the new attestation workflow. When the Attestation Agent receives an attestation request from the Verifier, it retrieves the current configuration from the GNSS receiver and parses the information read by the interface so that data acquires a well-established integrity semantics. Afterwards, the Attestation Agent writes the parsed configuration in the currentGNSSconfig.txt file and re-reads the same file to trigger a new measurement event in the IMA module. This process will produce a new entry in the IMA ML if the file has not yet been measured or its digest has changed from the previous measurements due to a manipulation of the receiver configuration. In this way, a Verifier is able to detect a violation that occurs in the configuration of the GNSS receiver by comparing the measure present in the IMA ML with a nominal measure, corresponding to the expected configuration previously inserted in the certified whitelist. To carry on the remaining process, the Attestation Agent creates an IR containing the TPM quote, the IMA ML and the AK credential, as described in Section 4.4, and sends the IR to the Verifier. The latter is now able to attest both the entire software stack of the Master Clock and the GNSS receiver configuration. Figure 7 summarizes all the steps performed by the Attestation Agent on the Master Clock node.

Entrusting the Attestation Agent with the task of monitoring the configuration of the GNSS receiver rather than a separate application has the advantage of ensuring a periodic check at each attestation cycle. Suppose we assign this task to a separate application, and this application inadvertently or maliciously stops—in that case, the Verifier may not be able to detect the incident and the fact that the receiver configuration monitoring has stopped. Instead, the single application approach allows the Verifier to detect the event in near-real time: stopping the Attestation Agent would trigger a timeout, thus, waiting for the IR on the Verifier side.

This attestation workflow can be straightforwardly extended to monitor the software configuration of other hardware devices installed on the platform, or standalone devices. Hardware devices are in fact very important components in the platform, since the system needs to communicate and exchange information with them during system boot and runtime, so they are a likely target of insidious and difficult-to-detect attacks by hardware adversaries [36]. Complete device security would need to provide hardware devices with a secure identity to perform authentication of the device and establish secure communication channels, thus, avoiding the modification of exchanged information, eavesdropping and masquerading; moreover, a complete measurement of the device firmware would be needed, including hardware and firmware configurations. However, our approach allows to easily enlarge the attestable surface of the platform without requiring any changes to the device firmware, which is often owned by the device vendor.

## 6. Implementation of the Solution

In this work, *Keylime* was chosen as the framework to perform the RA protocol. Keylime is an innovative open source project for monitoring the integrity of nodes deployed in distributed infrastructures via periodic remote attestation, leveraging TPM 2.0 and IMA technologies. MIT researchers developed Keylime as a solution able to make Trusted Computing technologies usable in cloud environments, allowing tenants to establish the trustworthiness of the cloud infrastructure and their own services running on it. This framework has been conceived as a software layer between trusted hardware- and software-based security services, and is currently one of the sandbox projects supported by the *Cloud Native Computing Foundation* (CNCF).

**Figure 8:**
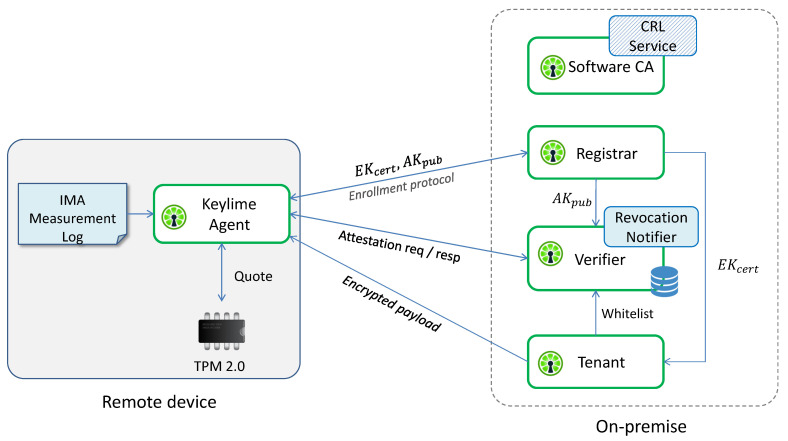
Simplified architecture of the Keylime framework.

Figure 8 represents a simplified architecture of the Keylime framework, which comprises the following components:

- the *Keylime Agent* is a service running on the remote platform; it performs the enrollment protocol with the Registrar by sending it the TPM credentials (EK_cert_ and AK_pub_) and responding to its challenge to demonstrate that AK is resident on the same TPM as EK; then, it waits for attestation requests, to which it responds with an IR containing a TPM quote and the IMA ML;
- the *Registrar* stores the TPM credentials received from the Keylime Agent and sends them to the Verifier and Tenant, allowing them to verify the authenticity of the TPM quotes and the EK certificate;
- the *Tenant* is the component that initiates the framework; it sends to the Keylime Agent an encrypted payload, typically containing identity keys, certificates and scripts used for handling revocation events; then, it registers the Keylime Agent to the Verifier, providing it the whitelist, the TPM policy (i.e., the list of PCRs) and other information needed to attest the integrity status of the platform; finally, it verifies the authenticity of the TPM installed on the remote device by checking the validity of the EK certificate;
- the *Verifier* is the core component of the Keylime architecture, since it is responsible for assessing the trustworthiness level of the remote device; it periodically sends attestation requests to the Keylime Agent, with a frequency that can be configured using a specific parameter, and verifies the IRs on the basis of the whitelist and TPM policy received from the Tenant and the AK_pub_ received from the Registrar;
- the *Software CA* is a certification authority whose goal is to link Trusted Computing functionalities with higher-level security services; if used for creating the certificates related to software identity keys of the nodes monitored by the Verifier, it will revoke the certificates as soon as the nodes become untrusted by publishing a new Certificate Revocation List (CRL);
- the *Revocation Notifier* completes the link between Trusted Computing and higher-level security services. When the Verifier detects an untrusted node, the Revocation Notifier sends a “revocation event” to the Software CA and the Keylime Agents that are registered to this service. Upon receipt of this event, the Software CA will update its CRL, while the Keylime Agents will execute the specific scripts received in the encrypted payload or those configured on the nodes, thus, allowing higher-level security services to automatically react to failed attestation events by ring-fencing the untrusted nodes; for example, by closing all TLS connections and VPN tunnels, or updating IP tables.

In order to support the solution presented in Section 4.4, we have introduced changes in the functioning of the Keylime Agent, whose behaviour is customizable through the parameters present in the configuration file keylime.conf. We defined two additional parameters related to this component:

- check_GNSS_config, a boolean that will be set to True on the Master Clocks and to False on the Slave Clocks;
- GNSS, a string that specifies the path to the character special file representing the GNSS receiver.

Moreover, we added a module to the framework, checkGNSSconfig.py, containing the functions related to the GNSS receiver. The check on the configuration of the GNSS module is activated depending on the values assigned to two new parameters. In particular, if check_GNSS_config is True and the path assigned to GNSS corresponds to a character special file in the system, the Keylime Agent, upon receipt of an attestation request, first monitors the receiver configuration (as described in Section 4.4) by invoking a function in the checkGNSSconfig.py module, and then creates and sends back the Integrity Report to the Verifier, as this normally happens in the Keylime framework.

Furthermore, it is possible to configure the framework in such a way that the nodes of the time distribution network rely on the service offered by the Revocation Notifier to automatically switch to the backup Master Clock in case the Grand Master Clock is compromised. For this purpose, it is necessary to define some scripts that contain the instructions to modify the configuration of the Master Clock used in the network. The scripts have to be sent to the nodes in the encrypted payload or loaded on the nodes and configured in the Keylime.conf file. These scripts will be automatically executed as soon as the Keylime Agents receive an authentic revocation event from the Revocation Notifier as a result of an integrity failure of the Master Clock.

## 7. Testbed Setting

This section provides a description of the experimental testbed used in this work, that represents a modified/extended version of the setup previously presented in [9]. This testbed is representative of a typical synchronization distribution network and is characterized by high flexibility, configurability, and scalability, being suitable to emulate different network topologies, but avoiding the complexities related to the operation of real PTP hardware. In addition, it is suitable to analyze selected attack vectors and countermeasures in a legal and controlled framework. The following paragraphs highlight and describe the main hardware and software components adopted in the testbed.

### 7.1. Hardware Components

The hardware setup used in this work includes five nodes in total, implemented by the means of the following components:

- a desktop PC Dell Precision 3440 (Intel Core i7-10700 CPU 2.9 GHz, 16 GB RAM, 512 GB HDD), that acts as the Verifier in the RA workflow;
- Raspberry Pi^®^ 4 (RPi4) Model B [37], a flexible and high-performance Single Board Computer with a well-supported set of software libraries and tools for Linux;
- mosaicHAT [38], an open source hat compatible with RPi4. It is based on the Septentrio’s mosaic-X5^®^ receiver [39], a multi-band, multi-constellation GNSS module representative of the state of the art (i.e., supporting newest Galileo signals, including the Open Service Navigation Message Authentication-OSNMA [40,41]);
- Infineon OPTIGA™ TPM SLI 9670 Iridium board [42], an evaluation board with the widely used TPM2.0 chip.

**Figure 9:**
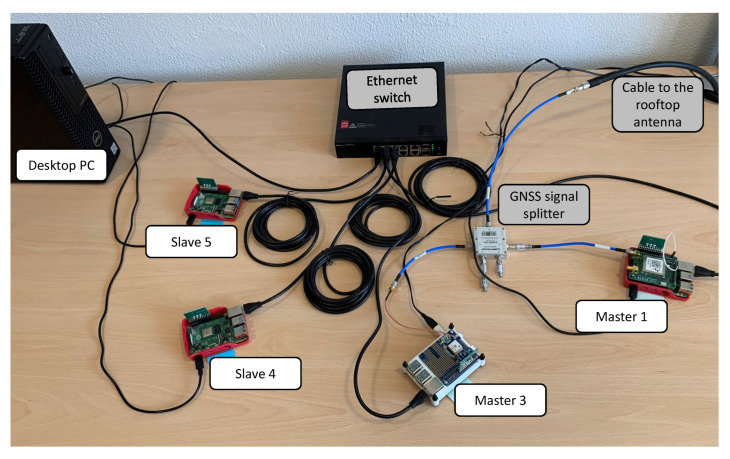
Picture of the full testbed, configured with two Master nodes and two Slave nodes.

In detail, Figure 9 shows the network topology that we have adopted in all the tests reported in this paper. The picture presents the following components highlighted with proper labels, counter-clockwise, starting from the upper-left corner:

- the desktop PC, used as Verifier for the RA workflow;
- two RPi4 configured as Slave nodes (i.e., Slave 4 and 5) with the TPM only, without a GNSS module;
- two RPi4 configured as Master nodes (i.e., labeled as Master 1 and 3, respectively), including both a GNSS module and a TPM stacked on top of it. For the sake of simplicity, this setup adopts two Master nodes instead of three, as in [9] (i.e., without previous Master 2). Only the Master 1 is equipped with the mosaicHAT [38], while the Master 3 has a low-cost GNSS module (i.e., Adafruit Ultimate GPS HAT [43]).

The desktop PC and all the nodes in Figure 9 are connected to the power supply and to the same Local Area Network (LAN) by the means of an Ethernet switch and cables, as shown in Figure 9. As far as the two Master nodes are concerned, we connected them through a GNSS signal splitter to the same antenna, which is a professional GNSS antenna (i.e., Tallysman^®^ VSP6037L VeroStar™ Full GNSS Precision Antenna plus L-band [44]), properly mounted on the rooftop of the building during the tests (only the cable to the rooftop antenna is shown in Figure 9).

### 7.2. Software Configuration and Attacks Emulation

We installed and properly configured the Keylime framework on the testbed in order to test and assess the proposed RA workflow. In detail, the desktop PC shown in Figure 9 executes the Keylime Verifier, Tenant and Registrar in order to attest the other four nodes, thus, running the Keylime Agent.

Moreover, the Master 1 is configured as the Grand Master clock for the PTP protocol. On the other hand, the Master 3 (i.e., the white node in Figure 9) acts as a backup clock, also used as a reference/monitoring node: it is capable to estimate the relative synchronization errors of the other nodes on the LAN with respect to its reference/local clock, taking advantage of the ntpq utility [45], and to collect detailed log files, by the means of a properly configured ntpd daemon [34].

As highlighted in Section 1, the following tests focus on “openbox” hardware attacks capable of compromising the configuration set on the GNSS receiver. In detail, we emulate specific attacks against the configuration of the GNSS receiver module installed on the Master 1 node (i.e., mosaic-X5), while the configuration of the Master 3 (reference) is never modified. It is assumed that, through privilege escalation, an attacker has gained access to the command interface (i.e., serial interface) of the mosaic-X5 GNSS receiver, and is able to modify the calibration parameters of the receiver. In practice, the attack consists of sending a malicious configuration command (i.e., setPPSParameters) to the mosaic-X5 receiver on the Master 1 node, with a wrong calibration parameter for its 1PPS signal output (i.e., a wrong cable delay). According to the mosaic-X5 Reference Guide [46], this parameter can be set in a range from −1 ms to +1 ms, thus, potentially resulting in an unacceptable offset with respect to the true time scale in a 5G synchronization network.

In this way, we are able to emulate a **basic attack**, consisting of a single modification on the GNSS receiver configuration (i.e., a time step) during the test duration.

**Figure 10:**
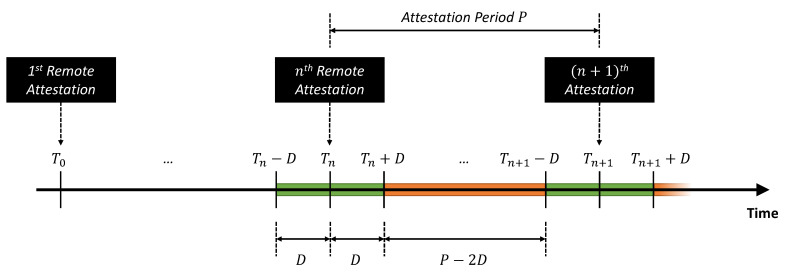
Illustration of the advanced attack scenario.

Apart from this simple case, we can also emulate **advanced attack** scenarios, as shown in Figure 10. In this case, the attacker is assumed capable of estimating the time instants where the RA protocol is actually executed (i.e., the Attestation Period $P=T_{n+1}-T_{n}$). The attacker can try to avoid the detection by the Keylime Verifier by dynamically adapting its attack strategy: they can repetitively misconfigure and reconfigure the GNSS receiver with a sufficient delay (i.e., *D*) with respect to each attestation period. As highlighted by the green boxes in Figure 10, the attacker leaves the GNSS receiver with its correct/nominal configuration for a duration of 2D around each repetition of the RA protocol, while the malicious configuration is introduced for an interval equal to (P−2D) during each attestation period (highlighted in orange in Figure 10). In this sense, it is possible to define an attack duty cycle parameter $A_{D}$ as the percentage of time where the GNSS receiver is actually misconfigured during each attestation period:

(2) $$A_{D}=\frac{P-2D}{P}\cdot 100=\left(1-\frac{2D}{P}\right)\cdot 100$$

## 8. Analysis of the Experimental Results

**Figure 11:**
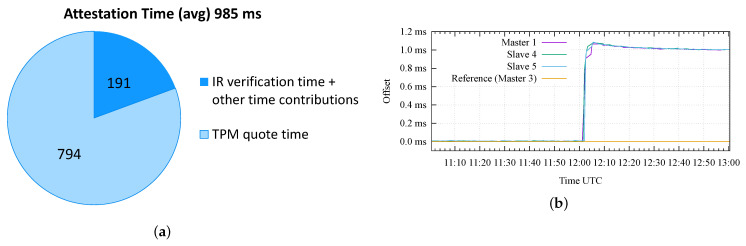
Obtained results for (**a**) assessment of the attestation time and (**b**) basic attack scenario.

Figure 11a presents the results of a preliminary analysis of the attestation time on the testbed. This analysis aims to assess the performance of the proposed RA workflow in order to identify the minimum achievable attestation period $P_{min}$ on this testbed. The figure shows that the average time taken by the framework to complete an attestation cycle, corresponding to $P_{min}$, is 984 ms ≈ 1 s; it is possible to observe that most of this time, 794 ms ≈ 0.8 s, is used for the creation of the quote, while 191 ms ≈ 0.2 s is used for the transmission of messages on the network and for the verification of the IR by the Verifier. These times were averaged over 894 attestations performed on the Master Clock.

After this initial analysis, we have emulated the attack scenarios introduced in the previous section. For all tests, we adopted a standard execution procedure, with a total duration of 2 h for each test; in the first hour, we measured the synchronization offset of all the nodes in their nominal configuration (i.e., not under attack), while in the second hour, we emulated a specific attack scenario, targeting the Master 1 node.

Figure 11b shows the obtained results in the case of the basic attack. In this case, the first hour of the test (from 11:00 to 12:00) shows a good synchronization accuracy and precision achieved between all the nodes of the testbed, with a negligible time offset and jitter. At 12:01, we performed the basic attack, introducing a time offset of 1 millisecond on the Master 1 with respect to the time reference (i.e., Master 3). It can be appreciated how the Slave 4 and 5 nodes follow the wrong time information distributed by the Master 1 and quickly reach a stable offset of 1 ms. Obviously, Keylime correctly detects the modified GNSS configuration on the Master 1 according to the selected attestation period; in this test, we configured a value of P=2 s, thus, resulting in detection within 2 s after 12:01.

**Figure 12:**
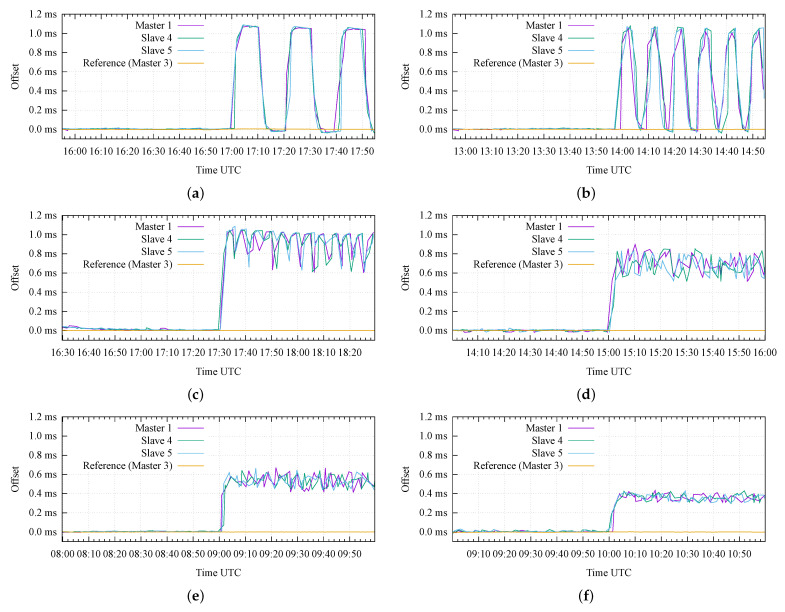
Results of advanced attack scenarios: (**a**) P=1200 s, D=300 s, $A_{D}=50$%; (**b**) P=600 s, D=150 s, $A_{D}=50$%; (**c**) P=300 s, D=15 s, $A_{D}=90$%; (**d**) P=90 s, D=15 s, $A_{D}=66.7$%; (**e**) P=60 s, D=15 s, $A_{D}=50$%; (**f**) P=45 s, D=15 s, $A_{D}=33.3$%.

Figure 12 presents the results of the advanced attack scenario, where we emulated the case of an attacker that tries to avoid the detection from Keylime by repetitively misconfiguring and re-configuring the GNSS receiver on the Master 1. We emulated this scenario under six different sets of configuration parameters, starting from a very large attestation period (P=1200 s, i.e., 20 min) in Figure 12a, and decreasing it to less than 1 min (i.e., P=45 s) in Figure 12f. In addition, we also changed the value of the attack delay (*D*), resulting in different attack duty cycle values ($A_{D}$).

Figure 12a corresponds to the case where the RA protocol is repeated every 20 min and the attack duty cycle is $A_{D}=50$%. In this case, the attacker repetitively introduces an offset of 1 ms for 10 min and then restores the nominal configuration (i.e., zero offset) in the following 10 min; this results in a (smoothed) square-wave pattern, clearly shown in Figure 12a for the Master 1 and the two Slave nodes.

On the other hand, Figure 12b corresponds to a case with the same duty cycle ($A_{D}=50$%), but with a shorter period (i.e., the attacker misconfigures and reconfigures the receiver with a 5 min granularity): it can be appreciated how the duration of each attack repetition starts to become comparable to the time required by the nodes to synchronize and stabilize their internal clocks, thus, resulting in a sawtooth-like pattern for the measured offsets.

A further decrease in the attestation period results in noisy patterns, with a remarkable jitter on the relative offsets between the nodes under attack, as can be appreciated in Figure 12c–f. In detail, these figures correspond to decreasing duty cycle values (i.e., $A_{D}=90$%, 66.7%, 50%, and 33.3%, respectively). The last three figures show that the measured offset values do not achieve the expected maximum value (i.e., 1 ms), but they seem to stabilize around values proportional to the duty cycle (i.e., 0.667 ms, 0.5 ms, and 0.333 ms, respectively).

From the results in Figure 12, it is possible to conclude that:

- In order to limit the potential impact of an advanced attack, the attestation period (*P*) must be configured to the lowest possible value (e.g., less than 1 min). Obviously, this design parameter typically results from a trade-off between security requirements and complexity (i.e., a larger attestation period can be desirable to limit the use of computational and communication resources);
- In addition, a randomization of the attestation period could be another potential solution in order to limit the capability of the attacker to estimate the beginning of each attestation period;
- Finally, a secure communication channel between the GNSS receiver module and the CPU of the node is another viable solution to solve this category of attack, so that the GNSS receiver would only execute authorized commands.

## 9. Conclusions and Future Work

In this work, we presented a solution capable of attesting the integrity of all components of the Master and Slave nodes available in a time synchronization network. The RA procedure that we propose can detect both a violation of the host system and of the GNSS receiver configuration. However, it is worth noticing that hardware attacks against the GNSS module are still possible since the communication channel between the receiver and the CPU package on the board is not authenticated. Just as common infrastructural nodes need to create secure channels in order to communicate securely, in the same way, a platform should have secure communication channels between all the end-points on its motherboard. Moreover, the communication between the host system and the hardware devices on the board requires mechanisms that allow both the authentication and the integrity of the device firmware (immutable ROM, mutable firmware, hardware configuration, firmware configuration). This allows the prevention of unauthorized hardware modifications and undesired firmware updates. The platform may also save firmware measurements in the TPM chip by allowing a remote entity to perform a full platform attestation [36]. For this reason, we believe that it is essential to enhance the security of a time distribution network, creating a mutually authenticated channel between the GNSS receiver and the host system, capable of resisting hardware adversaries that aim to compromise data integrity and confidentiality, and enabling the measurement flow of the receiver’s hardware and firmware identity for ascertaining its trustworthiness.

Furthermore, since the nodes of a time distribution network have a static configuration for an extended period of time, future work might exploit the Implicit Attestation [47] technique for the creation of “trusted” communication channels between the Master Clock and the Slave Clock nodes to create a simplified attestation scheme which does not require the presence of a centralized entity, such as the Verifier.

## Data Availability

Not Applicable.

## Abbreviations

The following abbreviations are used in this manuscript:

1PPS	1 Pulse-Per-Second
5G	Fifth Generation of cellular network
AK	Attestation Key
CA	Certificate Authority
CNCF	Cloud Native Computing Foundation
CPU	Central Processing Unit
CRL	Certificate Revocation List
CRTM	Core Root of Trust for Measurement
DoS	Denial of Service
EK	Endorsement Key
GNSS	Global Navigation Satellite System
IAK	Initial Attestation Key
IEEE	Institute of Electrical and Electronics Engineers
IMA	Integrity Measurement Architecture
IP	Internet Protocol
IR	Integrity Report
LAK	Local Attestation Key
LAN	Local Area Network
LINKS	Leading Innovation & Knowledge for Society
LTE-FDD	Long-Term Evolution-Frequency Division Duplex
MDPI	Multidisciplinary Digital Publishing Institute
MITM	Man-In-The-Middle
ML	Measurement Log
NMEA	National Marine Electronics Association
NTS	Network Time Security
OSNMA	Open Service Navigation Message Authentication
PCR	Platform Configuration Register
PTP	Precision Time Protocol
PVT	Position, Velocity and Time
RA	Remote Attestation
RAN	Radio Access Network
RF	Radio-Frequency
ROOT	Rolling Out OSNMA for the secure synchronization of Telecom networks
RoTs	Roots of Trust
RTM	Root of Trust for Measurement
RTR	Root of Trust for Reporting
RTS	Root of Trust for Storage
SoC	System on Chip
TCB	Trusted Computing Base
TCG	Trusted Computing Group
TDD	Time Division Duplex
TLS	Transport Layer Security
TP	Trusted Platform
TPM	Trusted Platform Module
VPN	Virtual Private Network
WR-PTP	White Rabbit extension of Precision Time Protocol

## References

[1] Falletti E., Margaria D., Marucco G., Motella B., Nicola M., Pini M. (2019). Synchronization of Critical Infrastructures Dependent Upon GNSS: Current Vulnerabilities and Protection Provided by New Signals. IEEE Syst. J..

[2] Pini M., Falletti E., Nicola M., Margaria D., Marucco G. Dependancy of power grids to satellite-derived time: Vulnerabilities and new protections. Proceedings of the 2018 IEEE International Telecommunications Energy Conference (INTELEC).

[3] Pini M., Minetto A., Vesco A., Berbecaru D., Contreras Murillo L.M., Nemry P., De Francesca I., Rat B., Callewaert K. Satellite-derived Time for Enhanced Telecom Networks Synchronization: The ROOT Project. Proceedings of the 2021 IEEE 8th International Workshop on Metrology for AeroSpace (MetroAeroSpace).

[4] Council of the European Union, Brussels, Belgium (2008). Council Directive 2008/114/EC of 8 December 2008 on the Identification and Designation of European Critical Infrastructures and the Assessment of the Need to Improve Their Protection. https://eur-lex.europa.eu/eli/dir/2008/114/oj.

[5] Boyle K. 5G Is All in the Timing. https://www.ericsson.com/en/blog/2019/8/what-you-need-to-know-about-timing-and-sync-in-5G-transport-networks.

[6] DeCusatis C., Lynch R.M., Kluge W., Houston J., Wojciak P.A., Guendert S. (2020). Impact of Cyberattacks on Precision Time Protocol. IEEE Trans. Instrum. Meas..

[7] Dovis F. (2015). GNSS Interference Threats and Countermeasures.

[8] Margaria D., Motella B., Anghileri M., Floch J., Fernandez-Hernandez I., Paonni M. (2017). Signal Structure-Based Authentication for Civil GNSSs: Recent Solutions and Perspectives. IEEE Signal Process. Mag..

[9] Margaria D., Vesco A. (2021). Trusted GNSS-Based Time Synchronization for Industry 4.0 Applications. Appl. Sci..

[10] Jiang Y., Wu S., Yang H., Luo H., Chen Z., Yin S., Kaynak O. (2022). Secure Data Transmission and Trustworthiness Judgement Approaches Against Cyber-Physical Attacks in an Integrated Data-Driven Framework. IEEE Trans. Syst. Man Cybern. Syst..

[11] Ren Y., Liu W., Liu A., Wang T., Li A. (2022). A privacy-protected intelligent crowdsourcing application of IoT based on the reinforcement learning. Future Gener. Comput. Syst..

[12] Guo J., Wang H., Liu W., Huang G., Gui J., Zhang S. (2021). A lightweight verifiable trust based data collection approach for sensor–cloud systems. J. Syst. Archit..

[13] Mo W., Wang T., Zhang S., Zhang J. (2020). An active and verifiable trust evaluation approach for edge computing. J. Cloud Comput..

[14] Bacci G., Falletti E., Fernández-Prades C., Luise M., Margaria D., Zanier F., Dardari D., Falletti E., Luise M. (2012). Chapter 2-Satellite-Based Navigation Systems. Satellite and Terrestrial Radio Positioning Techniques.

[15] Dovis F., Margaria D., Mulassano P., Dominici F. (2018). Chapter 20—Overview of Global Positioning Systems. Handbook of Position Location.

[16] (2008). IEEE Standard for a Precision Clock Synchronization Protocol for Networked Measurement and Control Systems.

[17] (2020). IEEE Standard for a Precision Clock Synchronization Protocol for Networked Measurement and Control Systems.

[18] Girela-López F., López-Jiménez J., Jiménez-López M., Rodríguez R., Ros E., Díaz J. (2020). IEEE 1588 High Accuracy Default Profile: Applications and Challenges. IEEE Access.

[19] Lipiński M., Włostowski T., Serrano J., Alvarez P. White rabbit: A PTP application for robust sub-nanosecond synchronization. Proceedings of the 2011 IEEE International Symposium on Precision Clock Synchronization for Measurement, Control and Communication.

[20] Pini M., Minetto A., Nemry P., Rat B., Contreras Murillo L.M., De Francesca I., Margaria D., Vesco A., Berbecaru D., Callewaert K. Protection of GNSS-based Synchronization in Communication Networks: The ROOT project. Proceedings of the European Navigation Conference & International Navigation Conference (Navigation 2021).

[21] Arnold D., Langer M. Adapting NTS to PTP. Proceedings of the 2020 International Timing and Sync Forum (ITSF).

[22] PaX Team Address Space Layout Randomization (ASLR). https://pax.grsecurity.net/docs/aslr.txt.

[23] Trusted Computing Group (2019). Trusted Platform Module Library, Part 1: Architecture, Specification, Family 2.0, Level 00, Revision 01.59. https://trustedcomputinggroup.org/wp-content/uploads/TCG_TPM2_r1p59_Part1_Architecture_pub.pdf.

[24] Challener D., Yoder K., Catherman R., Safford D., Doom L.V. (2007). A Practical Guide to Trusted Computing.

[25] Pedone I., Canavese D., Lioy A., Vacca J.R. (2020). Trusted Computing Technology and Proposals for Resolving Cloud Computing Security Problems. Cloud Computing Security: Foundations and Challenges.

[26] Trusted Computing Group (2019). TPM 2.0 Library. https://trustedcomputinggroup.org/resource/tpm-library-specification/.

[27] Arthur W., Challener D. (2015). A Practical Guide to TPM 2.0.

[28] Trusted Computing Group (2020). TCG Algorithm Registry.

[29] Integrity Measurement Architecture (IMA). https://sourceforge.net/p/linux-ima/wiki/Home/.

[30] Sfyrakis I., Gross T. (2020). A Survey on Hardware Approaches for Remote Attestation in Network Infrastructures. arXiv.

[31] Trusted Computing Group (2019). TCG Trusted Attestation Protocol Information Model. https://trustedcomputinggroup.org/resource/tcg-tap-information-model/.

[32] Trusted Computing Group (2011). TCG Infrastructure Working Group Integrity Report Schema. https://trustedcomputinggroup.org/wp-content/uploads/IWG_Integrity_Report_Schema_v2.0.r5.pdf.

[33] National Marine Electronics Association (2018). NMEA 0183 Interface Standard, Version 4.11. https://www.nmea.org/content/STANDARDS/NMEA_0183_Standard.

[34] The NTP (R&D) Project (2022). ntpd-Network Time Protocol (NTP) Daemon. http://doc.ntp.org/documentation/4.2.8-series/ntpd/.

[35] Owczarek W., Kreuzer S., Neville-Neil G.V. (2019). PTPd Official Source- Precision Time Protocol Daemon (1588-2008). https://github.com/ptpd/ptpd.

[36] Yao J., Zimmer V. (2020). Building Secure Firmware.

[37] Raspberry Pi® Trading Ltd (2021). Raspberry Pi® 4 Computer Model B, Product Brief. https://datasheets.raspberrypi.org/rpi4/raspberry-pi-4-product-brief.pdf.

[38] Sa’d J. (2020). MosaicHAT: An Open Source Raspberry Pi HAT Based on Septentrio’s Mosaic-X5. https://github.com/septentrio-gnss/mosaicHAT.

[39] Septentrio NV (2021). Mosaic-X5®: Compact, Multi-Constellation GNSS Receiver Module. https://www.septentrio.com/en/products/gnss-receivers/rover-base-receivers/receivers-module/mosaic.

[40] European Union Agency for the Space Programme (2021). Galileo Open Service Navigation Message Authentication (OSNMA) Info Note. https://www.gsc-europa.eu/sites/default/files/sites/all/files/Galileo_OSNMA_Info_Note.pdf.

[41] Septentrio NV (2022). Septentrio Brings OSNMA Anti-Spoofing Security to Mmarket. https://www.septentrio.com/en/company/news/septentrio-brings-osnma-anti-spoofing-security-market.

[42] Infineon Technologies AG (2019). OPTIGA™ TPM Application Note. Integration of an OPTIGA™ TPM SLx 9670 TPM2.0 with SPI Interface in a Raspberry Pi® 4 Linux Environment. https://www.infineon.com/dgdl/Infineon-OPTIGA_SLx_9670_TPM_2.0_Pi_4-ApplicationNotesv07_19-EN.pdf?fileId=5546d4626c1f3dc3016c3d19f43972eb.

[43] Adafruit Industries (2021). Ultimate GPS HAT for Raspberry Pi. https://cdn-learn.adafruit.com/downloads/pdf/adafruit-ultimate-gps-hat-for-raspberry-pi.pdf?timestamp=1627027424.

[44] Tallysman® (2021). VSP6037L VeroStar™ Full GNSS Precision Antenna Plus L-Band. https://www.tallysman.com/product/vsp6037l-verostar-full-gnss-antenna-l-band/.

[45] The NTP (R&D) Project (2022). ntpq-Standard NTP Query Program. https://doc.ntp.org/documentation/4.2.8-series/ntpq/.

[46] Septentrio N.V (2020). Mosaic-X5® Reference Guide, version 4.8.2.

[47] Trusted Computing Group (2019). TCG Trusted Attestation Protocol (TAP) Information Model for TPM Families 1.2 and 2.0 and DICE Family 1.0. https://trustedcomputinggroup.org/wp-content/uploads/TNC_TAP_Information_Model_v1.00_r0.36-FINAL.pdf.

